# Drought-Induced Responses in Maize under Different Vapor Pressure Deficit Conditions

**DOI:** 10.3390/plants11202771

**Published:** 2022-10-19

**Authors:** Mura Jyostna Devi, Vangimalla R. Reddy, Dennis Timlin

**Affiliations:** 1 USDA-ARS, Adaptive Cropping Systems Laboratory, Beltsville, MD 20705, USA; 2 USDA-ARS, Vegetable Crops Research Unit, Madison, WI 53706, USA; 3 Department of Horticulture, University of Wisconsin-Madison, Madison, WI 53706, USA

**Keywords:** abscisic acid, drought, enzymes, gene expression, photosynthesis, stomatal conductance, vapor pressure deficit, water use efficiency

## Abstract

Water stress in plants depends on the soil water level and the evaporative demand. In this study, the physiological, biochemical, and molecular response of maize were examined under three evaporative demand conditions (low—1.00 kPa, medium—2.2 kPa, and high—4.00 kPa Vapor pressure deficit (VPD)) at three different soil water content (SWC); well-watered, 45%, and 35% SWC. Plants grown at 35% SWC under high VPD had significant (*p* < 0.01) lower leaf weight, leaf area, and leaf number than low VPD. Plants under low, medium, and high VPD with drought stress (45% and 35% SWC) showed a 30 to 60% reduction in their leaf area compared to well-watered plants. Gas exchange parameters including photosynthesis, stomatal conductance, and water use efficiency exhibited significant differences (*p* < 0.01) between treatments, with the highest reduction occuring at 35% SWC and high VPD. Both drought and VPD significantly (*p* < 0.01) increased C_4_ enzyme levels and some transcription factors with increased stress levels. Transcription factors primarily related to Abssisic Acid (ABA) synthesis were upregulated under drought, which might be related to high ABA levels. In summary, severe drought levels coupled with high VPD had shown a significant decrease in plant development by modifying enzymes, ABA, and transcription factors.

## 1. Introduction

Irrigation is critical for improving agricultural yield. Water use efficiency must be improved in order to produce more food with less water to meet future food demands for a growing population [1]. New cultivars developed through innovative biotechnology and conventional breeding approaches do not help achieve high yields if the water is limited [2]. Drought-related crop loss is common in maize-growing areas across the United States, even if it is not always visibly noticeable [3]. Water plays a predominant role in plant nutrient transport, chemical and enzymatic reactions, cell expansion, and transportation [4]. Water limitation results in anatomical and morphological modifications along with physiological and biochemical processes altering several plant functions [5,6]. It is crucial to study these physiological, biochemical, and molecular processes to improve water use efficiency in plants under water-limited conditions.

Plant water stress is determined by the soil water supply and the atmospheric evaporation rate [7]. Drought is often accompanied by elevated air and leaf temperatures; consequently, leaves experience additional evaporative demand due to increased leaf-to-air VPD [8]. Many factors are responsible for the yield loss due to water stress, such as the severity and duration of the stress, soil type, growth stage, plant species, and genotype [9]. Changes in evaporative demand can affect leaf growth even in the absence of a soil water deficit, but the magnitude and kinetics of these effects vary widely. Tardieu et al. [10] observed adverse effects of evaporative demand, as measured by the leaf-to-air vapor pressure difference (VPD), in well-watered field-grown maize during the day. Several studies found that high evaporative demand had a negative impact on leaf expansion, gas exchange, and anatomical features, along with changes in water transport genes and developmental genes [6,11,12].

Dry conditions during growth period will reduce plant and leaf size. Reduced leaf area will decrease transpiration and photosynthesis, thereby reducing crop yield. Minor reductions in the leaf area might have a small impact on yield, while a significant decrease in the leaf area could potentially reduce yields. The division and expansion at the cellular level in a plant’s growth zones determine organ and plant level growth responses to drought. Plants adapt to drought by avoiding dehydration through mechanisms such as stomatal closure, decreased cell growth, and photosynthesis, in addition to reducing leaf area [13]. Drought stress also causes a decrease in C_4_ enzymes that are involved in the Hatch-Slack pathway, including phosphoenolpyruvate carboxylase (PEPC), NADP-malic enzyme (NADP-ME), malate dehydrogenase (MDH), and pyruvate phosphate dikinase (PPDK) [14,15]. PEPC, NADP-ME, and/or PPDK have been implicated in certain types of stress responses, including osmotic stress [16]. Changes in the activities of the enzymes of the malate valves and expression levels of the MDH isoforms to abiotic stresses can be observed and play major roles in reductant export under stress conditions [17]. Even though these responses have been studied in maize subjected to only drought [15,18], little is known about their regulation under both drought and high evaporative demand. 

Many studies indicate that ABA (Abscissic acid), produced in the roots and transported to leaves via xylem, was primarily responsible for stomatal closure during soil drought [19,20]. Xie et al. [21] demonstrated that stomatal response to reduced atmospheric humidity was regulated through ABA mediated signaling. A few studies also indicated that leaf ABA metabolism is involved in response to evaporative demand [19]. Stomatal closure and also the expression of drought-stress related genes were induced by the phytohormone abscisic acid. Different transcription factor (TF) families have been identified as modulators of gene regulation in response to maize’s drought stress. The major stress regulating TFs such as MYB (Myeloblastosis), NAC (NAM, ATAF, and CUC), WRKY, bZIP (Basic leucine zipper domain), bHLH (Basic helix loop helix), dehydration-responsive element-binding protein (DREB), HD-zip play a significant role in stress tolerance through ABA-dependent or ABA-independent pathways in different crop species [22]. In maize, AP2/ERF (APETALA2/Ethylene Responsive Element Binding Factor), DREB, C_2_H_2_ ZF (Cys2His2 Zinc Finger), MYB and bHLH are identified as important TFs for drought tolerance [22]. Additionally, these transcription factors are involved in regulating various physiological and molecular functions stomatal regulation, hormone signaling, root development, and osmoregulation in maize [23]. However, no studies have observed the response of TF’s to drought and evaporative demand.

The purpose of this study was to determine the effects of drought stress on the growth and development of maize under different evaporative demand conditions. The second objective was to investigate the physiological, biochemical, and molecular levels’ response to water deficit under dry environmental conditions to enhance maize’s drought tolerance.

## 2. Results

In this study, the impact of drought on maize under varying evaporative demand levels was examined. Overall, two similar experiments were carried out, and a mean analysis was carried out for environmental parameters (Temperature, RH and PPFD) and physiological traits. There were no significant differences between the two experiments for physiological parameters and the environmental conditions. The data from both experiments was pooled for each physiological parameter, and the results are presented here. In the case of drought stress treatment, the soil was not watered until soil water content reached 45% (mild stress) and 35% SWC (severe stress). Once the soil water had reached SWC 45% and 35%, the SWC levels were maintained approximately at the same level gravimetrically. A significant decrease in leaf area, leaf weight, and leaf number were observed under high VPD conditions with water limitation compared to low and medium evaporative demand conditions. Similar effects can also be noticed in terms of photosynthesis and water use efficiency.

### 2.1. Leaf Traits

**Figure 1:**
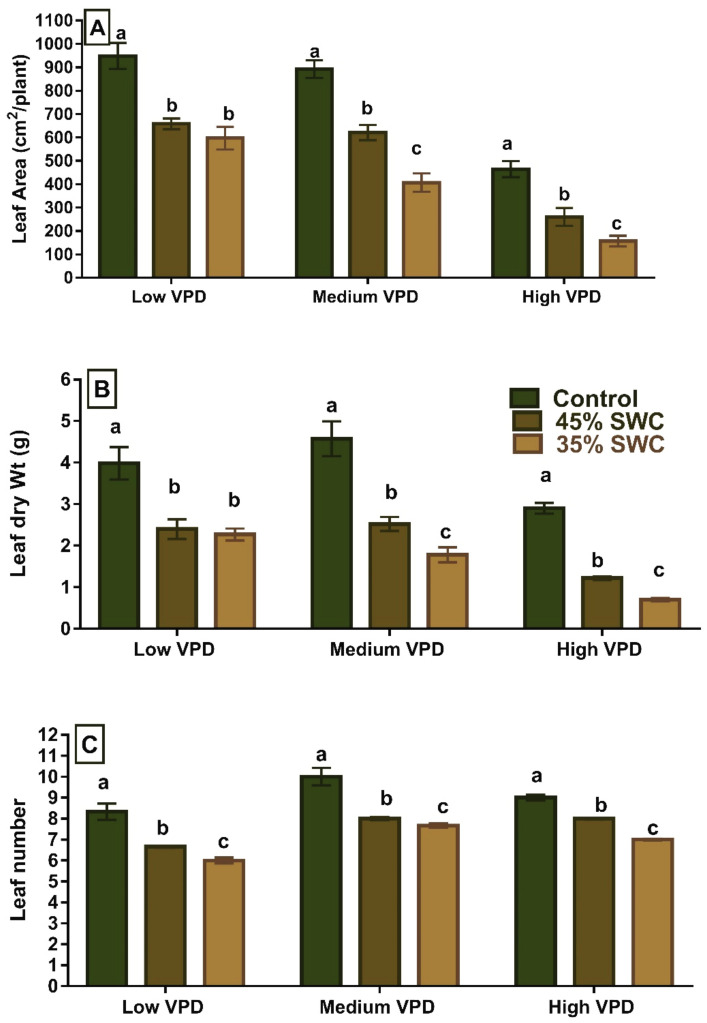
Average (**A**) Leaf area (cm^2^/plant) and (**B**) Leaf dry wt. (g/plant) and (**C**) Leaf number of maize under low, medium and high vapor pressure deficit (VPD) conditions to three different soil water levels (Well-Watered, 45% Soil water content (SWC), and 35% SWC). The bars (±average) represented by the same alphabet were not significant at *p* < 0.05 based on Tukey’s Kramer method at that particular VPD level.

A significant effect of drought on leaf area, leaf weight, and leaf numbers at low, medium, and high VPD levels was observed (*p* < 0.01). In comparison to the well-watered treatment, soil water content (45%) decreased the final leaf area by 30, 30, and 43.9% at low, medium, and high VPD levels, respectively. This was associated with a significant leaf dry weight and leaf number reduction by 39% and 50%, respectively, in low VPD environments. Under severe stress conditions (35% SWC), high VPD reduced the leaf area, weight, and leaf number by 30 to 60% (Figure 1 and Table 1). Under medium and high VPD conditions, there were significant differences in the leaf area, leaf dry weight, and leaf number across soil water stress treatments. However, at low VPD treatment, no differences in leaf area and leaf dry weight between 45% SWC and 35% SWC were observed (Figure 1). A significant percentage of reduction in leaf area, leaf weight, and leaf number relative to well-watered treatment was observed across VPD treatments (Figure 1 and Table 1).

### 2.2. Gas Exchange

**Figure 2:**
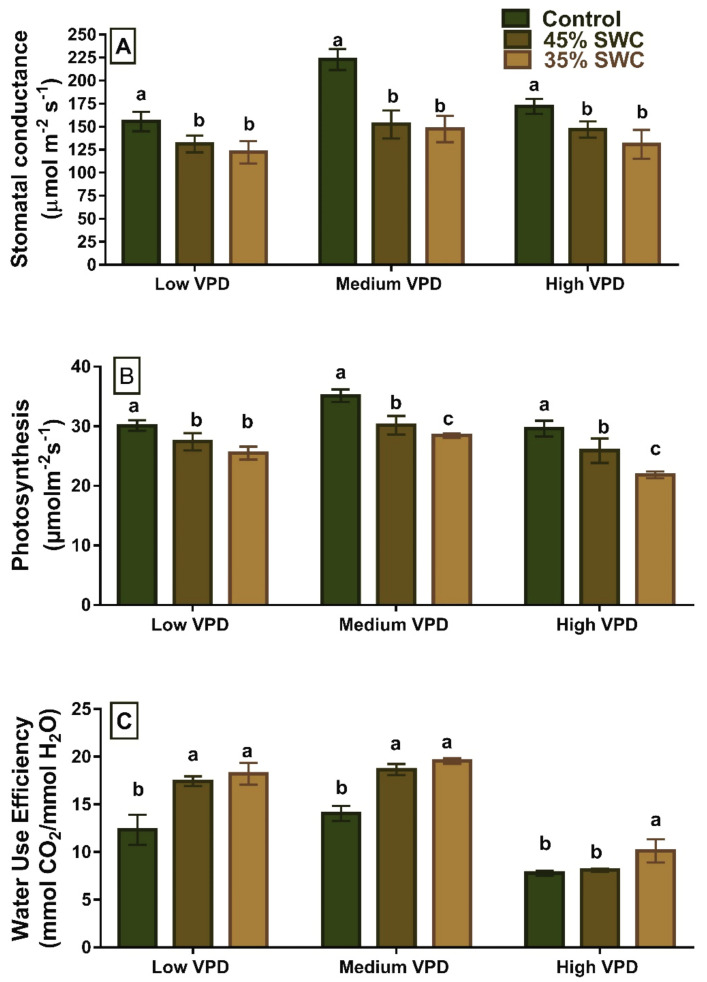
Gas exchange parameters (**A**) stomatal conductance (µmol m^−2^ s^−1^), (**B**) photosynthesis (µmol m^−2^ s^−1^), and (**C**) water use efficiency (mmol CO_2_/mmol H_2_O) of maize to different soil water content (SWC) well-watered, 45% SWC, and 35% SWC under low, medium and high vapor pressure deficit (VPD) conditions. The bars (Average ± S.E.) at each VPD represented with the same alphabet were not significantly different from each other at *p* < 0.05.

Drought and VPD had a significant interactive effect on all gas exchange measurements (*p* < 0.01). The maize response to 45% and 35% soil water content at low and high evaporative demand levels for all the gas exchange parameters was comparable (Figure 2, Table 1). In low, medium, and high evaporative demand conditions, there was a significant decrease in photosynthesis and stomatal conductance with an increase in the severity of drought stress. Plants at high VPD (4 kPa) exhibited significantly lower gs and A compared to low and medium VPD conditions (*p* < 0.001). However, a great percentage of reduction in the stomatal conductance was noticed at medium VPD level with 35% SWC (33.78%). Under all VPD conditions, a significant increase in water use efficiency was observed in 35% and 45% SWC treatments, with increases ranging from 4.04% to 60%. (Table 1).

### 2.3. Enzyme Activities

**Figure 3:**
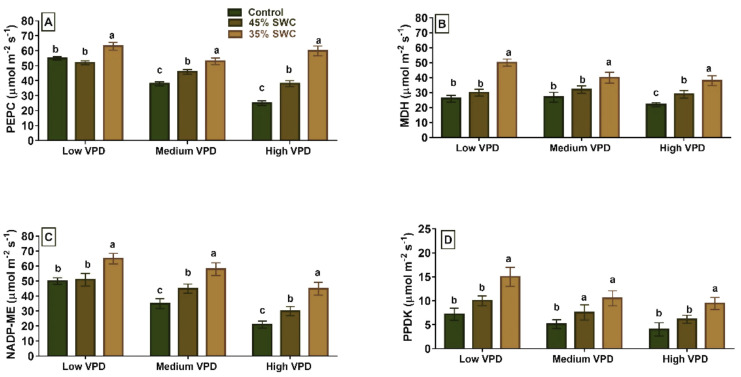
Enzymatic activity levels of four different enzymes (**A**) PEPC (**B**) MDH, (**C**) NADP-ME, and (**D**) PPDK (µmol m^−2^ s^−1^) in response to water limited stress (45% and 35% soil water content SWC) and evaporative demand (low, medium, and high vapor pressure deficit (VPD)). Bars (Average ± S.E.) represented with the same alphabet means the values were not significantly different at *p* < 0.05 calculated using Tukeys-Kramer.

We investigated the response of phosphoenolpyruvate carboxylase, malate dehydrogenase, NADP-malic enzyme, and pyruvate phosphate kinase in response to drought and VPD in maize. All four enzymes measured displayed significant differences across different water stress and VPD treatments (Figure 3). All enzymes showed a significant increase in activity levels at 45% and 35% SWC to all three VPD levels, except for PPDK at 45% SWC under medium VPD (Figure 3). Enzyme-specific activities in maize leaves of drought-stressed plants were significantly increased under high VPD conditions at 45% SWC (*p* < 0.01). The percentage increase over control plants was 52% for PEPC, 43% for NADP-ME, 31% for MDH, and 52% for PPDK (Table 1).

### 2.4. Foliar ABA

**Figure 4:**
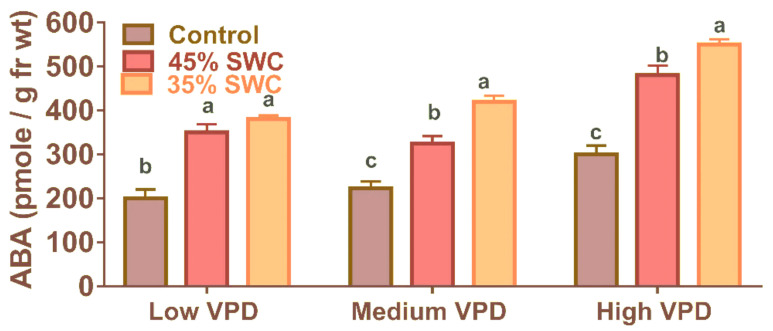
Foliar abscisic acid (ABA) (Pico mole/g fresh weight) concentration of maize subjected to three water treatment levels under three different vapor pressure deficit (VPD) levels. The bars (Average ± S.E.) represented by similar alphabet were not significantly different calculated based on Tukeys-Kramer method at *p* < 0.05.

ABA, like water use efficiency, was measured only from leaf samples collected in the second experiment. The concentration of foliar ABA was significant (*p* < 0.01) at three different VPD conditions, both in 45% and 35% SWC treatments except under low VPD (Figure 4). At 45% SWC, ABA levels increased significantly, ranging from 29.83 to 47.70% across all VPDs. Similarly, a significant percentage of the increase in ABA was observed at 35% SWC across various VPD treatments ranging from 83.3 to 90.0%. (Table 1).

### 2.5. Expression of Transcription Factors

**Figure 5:**
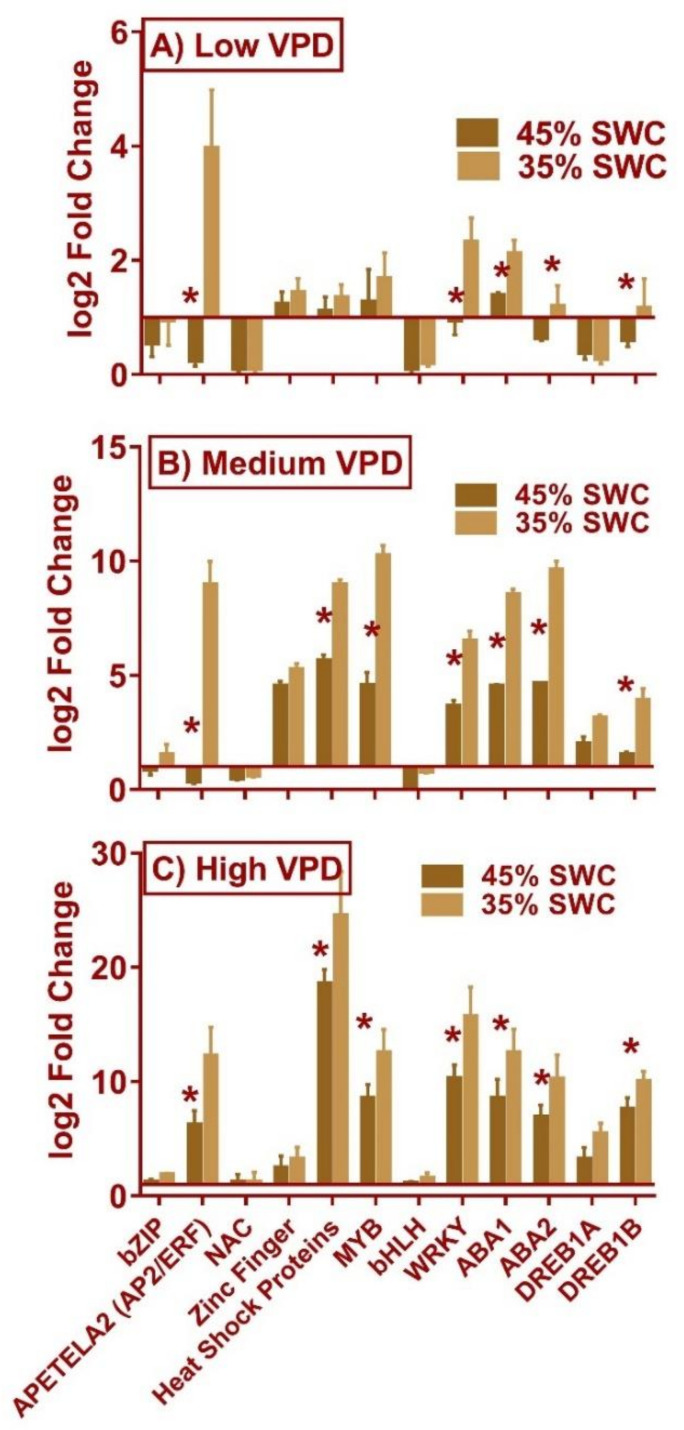
The relative gene expression of 12 different drought responsive transcripts measured in maize subjected to three soil water levels (well-watered, 45% soil water content (SWC), and 35% SWC) at three different vapor pressure deficit (VPD, kPa) conditions. The relative expression of 45% SWC and 35% SWC at each VPD level was calculated based on the expression in well-watered conditions. Bars (Average ± S.E.) represented with * were significantly different for 45% and 35% based on Tukey’s kramer method at each VPD level.

The changes in gene expression of 12 transcription factors (TF) in the samples collected from the second experiment were significant for some TF’s under three distinct VPD levels. The abundance of TFs was found to increase significantly along with the level of VPD (Figure 5). Not all TF’s displayed an increase in their abundance. The TF’s that showed increased/decreased their abundance in all treatments were APETELA2 (AP2/ERF), WRKY, ABA responsive binding factor 1, ABA responsive factor, and DREB1B. Zinc finger and heat shock protein factor (HSF) increased their expression levels only under medium and high VPD conditions (Figure 5).

## 3. Discussion

In the current study, significant interactive effects of drought and high evaporative demand on maize leaf development and gas exchange parameters related to TF expression and ABA were observed. The long-term exposure of maize to different VPD and water-limited stress showed a significant effect of high evaporative demand in the plants grown at 35% SWC compared to other treatments (Figure 1 and Figure 2). Drought stress and atmospheric VPD are the significant environmental variables affecting leaf traits and gas exchange parameters, and their effects have been observed extensively in many species [24,25]. In a recent study with maize, even without limitation in the soil water, high vapor pressure deficit reduced leaf expansion rate [6]. High VPD under water-limited conditions worsens the stress effects on plants either by increasing the TR (Transpiration Rate) or reducing carbon uptake. Even though high VPD generally increases the diffusion process, plants regulate transpiration through stomatal closure or employing other physiological responses [26]. This study observed a significant decrease in stomatal conductance and photosynthesis in the plants grown under medium VPD conditions than in high VPD, both at 45% and 35% SWC.

Plants with limited transpiration at high VPDs could not maintain the reduced transpiration at high VPDs obtained with high temperatures in a study with various maize cultivars [27]. The reduction in stomatal conductance and hence limitation in the transpiration can also be noticed at high VPD well-watered plants, which was not the case with medium VPD. The long-term exposure of maize plants at high VPD obtained with high temperature might have a negative effect on the stomatal conductance of well-watered, 45% SWC, and 35% SWC plants (Figure 2). The differences in aquaporin expression to different VPD’s at high temperatures also affect the leaf expansion rate and gas exchange measurements in maize [6]. This could be because the high temperature in the high VPD treatment affected the water channel proteins and membrane stability [28]. Even though the medium VPD treatment had a greater reduction in stomatal conductance at 45% and 35% SWC, the plants grown under high VPD conditions had a greater decrease in photosynthesis (Table 1). Plants minimize water loss and maintain plant cell hydration as VPD increases by reducing stomatal conductance in response to soil water deficit and low water vapor. Drought and high VPD, according to many studies, reduce stomatal conductance, affecting photosynthesis and growth [6,29,30,31,32]. The leaf number and weight reduction were observed under medium and high VPD conditions at 45% and 35% SWC (Table 1). It is evident that the soil water deficit and the high VPD impeded the plants’ growth. Under drought and high evaporative demand conditions, a reduction in both leaf area and leaf dry weight was observed in other studies [33]. The reduction in photosynthesis due to VPD and limited soil water content agrees with the previous studies. Plants grown at 35% SWC displayed a higher water use efficiency than 45% SWC plants. This agrees with the earlier study where plants improve water use efficiency under low soil moisture or high VPD or both stresses by limiting transpiration but maintaining some minimal photosynthesis [34].

In addition to modifying leaf characteristics and gas exchange parameters, plants adapt to conditions of drought stress and evaporative demand through biochemical mechanisms. Leaf enzyme analyses revealed that PPDK, PEPC, MDH, and NADP-ME activity were increased in response to elevated VPD across soil water stress treatments (Figure 3 and Table 1). In a previous maize study, photosynthesis and the activities of C_4_ enzymes, i.e., PPDK and NADP-ME, acclimated to growth temperatures [35]. PEPC, NADP-ME and PPDK are the key enzymes of C_4_ photosynthesis evolved to concentrate CO_2_ for the Calvin cycle especially in dry and hot environments. PEPC and/or NADP-ME and/or PPDK were reported to participate in some types of stress responses, including osmotic stress [16,36,37]. All enzymes showed a significant increase in activity under high evaporative stress and 35% SWC compared to mild stress (45% SWC). Similar results were observed in a tobacco study, where elevated levels of PEPC, NADP-ME, and PPDK were detected at both the enzymatic and transcript levels [36]. The increased activities of PEPC, NADP-ME, MDH, and PPDK are required to cope with higher amounts of reactive oxygen species produced during drought in order to reduce abiotic stress damage [17,36,38]. This study revealed that all these enzymes in maize responded to evaporative demand and limited water supply.

In general, the earliest response to drought is to reduce stomatal conductance to limit transpiration, which has been attributed to chemical ABA signaling playing an important role in controlling water flux in plants and reducing transpiration per leaf area [20]. An increase in the foliar ABA content as the drought progressed in all VPD treatments agrees with ABA’s role in the stomatal closure under drought, which was widely observed in several other studies (Figure 4) [19,20]. The potential ABA-producing tissues are located at various points along the continuum of soil–plant–atmosphere, and are therefore differentially sensitive to soil drought and evaporative demand [19,39]. Similar increases in ABA can also be observed with medium and low VPD treatments (Figure 4). However, the complexity of responses is largely determined by the stress threshold, and the effect of ABA chemical messengers, which varies across species and timescales [40]. In this study, a significant increase in the ABA at 35% SWC than 45% treatment or control demonstrated ABA involvement in the reduction in stomatal conductance and other parameters under stress conditions.

The response of gene transcription factors (TF’s) to drought and evaporative demand was observed in this research. Except for NAC, bHLH, and DREB1A, which were down regulated under low VPD at 45% and 35% SWC, most transcription factors assessed showed upregulation under stress conditions. Particularly, the increase in levels of APETELA2 (AP2/ERF), WRKY, MYB, ABA response element binding factor, ABA response element factor, and DREB1B as the stress level increased confirms the involvement of these TFs in the maize drought stress responses. Some of TF’s heat shock protein factors, WRKY, and DREB1B increased their expression level with increasing VPD level. In maize, AP2/ERF TFs can regulate a multitude of transcriptional programs by encoding different proteins to participate in a variety of stress responses. The upregulation of WRKY TF’s in maize conferred drought tolerance and protected membrane integrity [41]. In addition, the increase in ABA levels and the increase in ABA-related transcription factors (TFs), such as ABA response element binding factor1, ABA response element2, and DREB1B, demonstrated the involvement of ABA in mechanisms related to drought and evaporative demand, which may have resulted in decreased stomatal conductance. DREB1B, a dehydration-responsive transcription factor (TF), has been shown to have a dual function in *Arabidopsis*, regulating the responses to dehydration and heat stress [42], and DREB1A in drought [43].

Overall, this study showed significant interactive effects of soil water stress and evaporative demand on the maize leaf expansion and development. The impact of drought stress was intense under high evaporative demand conditions on leaf expansion, leaf weight, and gas exchange parameters. The enzyme analysis, ABA, and transcription factor analysis revealed that the maize physiological responses regulate via biochemical and molecular responses. The regulation of ABA TF’s and other TFs are related to physiological responses.

## 4. Materials and Methods

### 4.1. Plant Material and Growth Conditions

Maize (*Zea mays* L.) (Pioneer hybrid 34N43), a drought-tolerant variety (www.pioneer.com (accessed on 18 October 2022)), was selected for detailed characterization of maize response to VPD and drought. Overall, two experiments were conducted to study physiological, biochemical, and molecular responses to VPD and soil drying. The studies were carried out at the controlled environment facility, Beltsville agricultural research center, Beltsville, MD, USA. The range of temperature, RH, and VPD obtained in both experiments was listed in Supplementary Table S1. A mock experiment was conducted to estimate field capacity, the number of days to achieve 45% and 35% SWC in each chamber, and the amount of water required to maintain the SWC level. After planting, the days to reach 45% and 35% SWC were seven and ten days, respectively, with mock experiment. The hybrid’s seed was sown into pots containing a soil mix of sterilized sand and vermiculite (1:1 ratio). One-gallon pots made of plastic with 16.5 cm depth and 16.5 cm diameter at the top were used for the experiments. 

All plants were initially grown in a greenhouse maintained at 28 °C/21 °C (day/night) with no humidity control for six days to achieve equal germination. Pots were then moved to growth chambers to maintain low, medium, and high VPD levels. The photon flux density in the chambers was maintained at 1000 μmol m^−2^ s^−1^ for 14 h/day. Four replicates were maintained per each well-watered, 45% SWC, and 35% SWC treatments in experiment 1 and six replicate in experiment 2. The ABA, enzyme analysis, and gene expression studies samples were collected from experiment 2 and immediately stored at −80 °C.

### 4.2. Leaf Area, Weight, and Number

The experiments were terminated after 20 days of treatment, and the plants were harvested to measure leaf area and leaf weight. Leaf number was counted after the harvest, and the leaf area was measured using a leaf area meter (LI-COR, LI-3000, Lincoln, NE, USA). Harvested plants were dried in a forced-air oven at 70 °C for at least 72 h before determining the dry weights.

### 4.3. Gas Exchange

A portable photosynthesis system was used to calculate the net photosynthesis rate, photosynthesis, and water use efficiency (CIRAS 3). The CO_2_ concentration in the leaf chamber was kept constant at 400 mol mol^−1^, and the temperature was set to match that of the growth chamber. The measurements were carried out at a photon flux density of 1000 mol m^−2^ s^−1^. All parameters were measured inside the growth chambers around 10 a.m.

### 4.4. Analysis of Foliar ABA Content

Leaf samples (3 replicates/treatment) from all growth chambers were collected to measure ABA. The samples were immediately frozen in liquid nitrogen and stored at −80 °C for further analysis following sampling. The ethyl acetate fractionation technique was used for sample extraction and preparation as explained in [44,45]. The leaf sample (150 mg) was ground with 2 mL of 80% methanol containing butylated hydroxytoluene (0.001%, *w*/*v*). The extract was mechanically shaken overnight at 4 °C and centrifuged for 10 min at 14,000× *g*). The supernatant was passed through a Sep-Pak column (waters C-18 cartridge), and the clear extract was vacuum-evaporated. Samples were then resuspended in 4 mL of distilled water, acidified to pH 2.5 with 0.1 N HCI, and partitioned 3 times against ethyl acetate. This mixture was divided into aqueous and organic phases to separate free ABA from inert ABA conjugates. The ethyl acetate fraction was collected, evaporated, and the residue was dissolved in 500 µL of sample buffer. ABA was measured using an immunoassay kit from phytodetek (Phytodetek^®^ Immunoassay Kit for ABA, Agdia, IN, USA) following the protocol. 

### 4.5. Enzyme Extraction and Enzyme Activity Assays

Similar to ABA, leaf samples (3 replicates per treatment) were collected for enzyme extraction from all growth chambers. A total of 100 mg of fresh leaf tissue was used to prepare leaf enzyme extract in 0.6 mL of extraction buffer (50 mM Tris-HCl (pH 7.5), 10 mM MgCl_2_, 1 mM EDTA, 1% PVP-40, 5 mM Na-Pyruvate, 10% glycerol, 1 M leupeptin, 5 mM DTT) using a glass homogenizer at 0 °C. The extract was transferred to a 1.5 mL Eppendorf tube and centrifuged at 14,000× *g* for two to four minutes. The supernatant was transferred to a new tube and stored in liquid nitrogen until enzyme assays were conducted.

The activity of enzymes NADP-malate dehydrogenase (MDH), Phosphoenol pyruvate carboxylase (PEPC), NADP-malic enzyme (NADP-ME), and Pyruvate Pi dikinase (PPDK) was measured spectrophotometrically at 25 °C following the methods described in Maroco et al. [46] and Kim et al., 2007 [47]. NADP-MDH was measured in 1 mL solution containing 50 mM Tris–HCl (pH 8.0), 1 mM EDTA, 100 mM oxalacetic acid, 10 mM NADPH and 0.025 mL leaf extract. The enzyme actitivty was meaaured betwwen by setting activity range between 30 to 60 s at OD 340 nm and temperature 25 °C. OD decreases with time.

PEPC was measured in 1 mL solution containing 50 mM Tris–HCl (pH 8.0), 5 mM NaHCO_3_, 5 mM MgCl_2_, 10 mM NADH, 10 mM PEP (tricyclohexlamine salt), 1-unit malate dehydrogenase and 0.025 mL sample. The enzyme activity was measured between 30 to 60 s at 340 nm and 25 °C. NADP- Malic Enzyme (ME) was measured in 1 mL solution containing 50 mM Tris–HCl (pH 8.0), 5 mM EDTA, 22.5 mM MgCl_2_, 5 mM malic acid, 5 mM dithioerythritol, 0.5 mM NADP+ and 0.025 mL leaf extract. The enzyme activity was measured between 70 to 120 s at 340 nm and 25 °C. The reaction was initiated with the addition of 0.045 mL of MgCl_2_ and the activity was recorded. OD increases with time. PPDK was assayed in 1 mL solution containing 0.1 M Tris–HCl (pH 8.0), 10 mM MgCl_2_, 1 mM EDTA, 1.25 mL Na-pyruvate, 2.5 mM K_2_HPO_4_, 50 mM NaHCO_3_, 5 mM DTT, 0.2 mM NADPH, 1.25 mM ATP, 2 units malate dehydrogenase, 2 units PEP carboxylase and 0.025 mL sample. The enzyme activity was measured between 30 to 60 s at 340 nm and 25 °C. All measurements were performed using a Shimadzu model 2101 spectrophotometer operated in the kinetic mode. Enzyme activities were calculated from the rate of change in optical density at 340 nm and set activity region time as detailed above.

### 4.6. RNA Extraction and Real Time Quantitative PCR

In the second experiment, three replicate leaf samples were collected from drought stressed and control plants from all VPD treatments for RNA isolation to quantify twelve transcription factors. Supplementary Table S2 lists the names of 12 transcription factors. Trizol (Invitrogen) reagent was used to extract total RNA from the leaf. RNA was quantified using a NanoDrop 1000 after DNaseI treatment (Ambion) (Thermo Fisher, Waltham, MA, USA).

Genes EF-1 (elongation factor-1) and beta tubulin 7 (tub 7) of maize were used to normalize all values in the QRT-PCR assays. Primers for QRT-PCR were designed using Primer3 software. Primer sequences used in the study and their primer efficiencies were listed in Supplementary Table S2. First strand cDNA was synthesized with 2 µg of total RNA, oligo (dT)_20_ primers and SuperScript III RNase H reverse transcriptase from Invitrogen. The resultant cDNA was diluted 10-fold and was used as a template for real-time quantitative polymerase chain reaction (QPCR). Amplifications were performed with a model Mx3005P QPCR System plus Brilliant SYBR^®^ Green QPCR Master Mix (Stratagene, La Jolla, CA, USA). The amplification reactions consisted of a 1 min denaturing step at 95 °C, followed by 40 cycles at 95 °C for 30 s, 60 °C for 1 min, ending with a melting curve program at 72 °C for 30 s. Three replicate reactions per sample were used to ensure statistical significance. The RNA from each sample was analyzed simultaneously. Primer efficiency was determined as explained in [48]. Expression levels for all candidate genes were computed based on the stable expression level of the reference gene as described by Pfaffal, 2001 [34]. The expression levels of all transcription factors at 45% and 35% were calculated with relative to well-watered plant at each VPD treatments level.

### 4.7. Statistical Analysis

Since both experiments were carried out under identical VPD and drought conditions, the environmental parameters (Temperature, relative humidity, VPD and PPFD) were compared across two experiments with *t*-tests using GraphPad Prism 9. The physiological parameters from experiments 1 and 2 were also compared using a *t*-test. Due to the lack of significant differences between both experiments, the results were combined in order to gain a better understanding of the effects of drought in various evaporative demand environments. Leaf characteristics, gas exchange measurements, foliar ABA content, enzymes, and gene expression were analyzed using a one-way ANOVA analysis using GraphPad Prism 9. Tukey’s Kramer was used to test for statistical differences between treatments.

## Figures and Tables

**Table 1:** Percentage of decrease and increase in different parameters measured under low, medium, and high VPD conditions at 45% and 35% Soil water content. The percentage of decrease or increase was calculated as a difference from well-watered conditions at that particular VPD.

45% Soil Water Content			
**% of Decrease**	**Low VPD**	**Medium VPD**	**HighVPD**
**Leaf Area**	30.62	30.40	43.97
**Leaf Wt.**	39.69	44.86	57.93
**Leaf Number**	20.00	23.33	11.12
**Stomatal Conductance**	15.63	31.61	14.53
**Photosynthesis**	9.07	12.14	14.50
**35% Soil Water Content**			
**Leaf Area**	37.07	54.40	66.38
**Leaf Wt.**	42.96	61.05	75.86
**Leaf Number**	28.01	20.03	11.12
**Stomatal Conductance**	21.41	33.78	23.84
**Photosynthesis**	15.38	19.02	26.16
**45% Soil Water Content**			
**% of Increase**	**Low VPD**	**Medium VPD**	**High VPD**
**Water Use Efficiency**	41.35	32.87	4.04
**ABA**	47.70	39.31	29.83
**PEPC**	0.00	21.05	52.00
**MDH**	15.38	18.52	31.82
**NADP-ME**	2.00	28.57	42.86
**PPDK**	38.89	47.80	51.65
**35% Soil Water Content**			
**Water Use Efficiency**	75.00	45.74	60.00
**ABA**	90.00	88.34	83.33
**PEPC**	14.55	39.47	140.00
**MDH**	92.31	48.15	72.73
**NADP-ME**	30.00	65.71	114.29
**PPDK**	108.33	105.77	133.36

## Supplementary Materials

The following supporting information can be downloaded at: https://www.mdpi.com/article/10.3390/plants11202771/s1, Table S1: Average temperature (°C), RH (%) and calculated VPD (vapor pressure deficit, kPa) with ± S.E. maintained in three chambers (low, medium and high VPD levels) during experiment 1 and experiment 2.; Table S2: Genes name, accession number, forward number, reverse number, and primer efficiency of twelve drought responsive gene transcripts measured in response to drought and evaporative demand
