# Supplementary material for: Drought-Induced Responses in Maize under Different Vapor Pressure Deficit Conditions
Source: Plants (Basel). 2022 Oct 19;11(20):2771. doi: 10.3390/plants11202771 (PMC9611867; doi:10.3390/plants11202771)
Supplement: Supplementary file 1 [file plants-11-02771-s001.zip › plants-1911012-supplementary.pdf]

Supplemental file

Drought-induced responses in maize under different vapor pressure deficit conditions

Mura Jyostna Devi [1,2,3*], Vangimalla R Reddy [1] and Dennis Timlin [1]

[1] USDA-ARS, Adaptive Cropping Systems Laboratory, Beltsville, MD 20705, USA
[2] USDA-ARS; Vegetable Crops Research Unit, Madison, WI 53706, USA
[3] Dept. of Horticulture, University of Wisconsin-Madison, WI 53706, USA
* Correspondence: Jyostna.mura@usda.gov; Tel.: (+16082654745, MJD)

Received: 27 August 2022; Accepted: 18 October 2022; Published: 19 October 2022

Supplementary Table S1: Table S1: Average temperature (°C), RH (%) and calculated VPD (vapor pressure deficit, kPa) with ± S.E. maintained in three chambers (low, medium and high VPD levels) during experiment 1 and experiment 2.

**Treatment**	**Average temperature (°C)**	**Average RH (%)**	**Range of VPD (kPa)**
	**Experiment 1**		
Low VPD	21.8 ± 2.1	65.2 ± 5.3	0.9 to 1.2 kPa
Medium VPD	28.4 ± 1.8	48.1 ± 5.5	1.9 to 2.1 kPa
High VPD	35.2 ± 2.0	30.2 ± 4.3	3.8 to 4.0 kPa
**Experiment 2**			
Low VPD	21.5 ± 1.9	64.2 ± 4.8	0.9 to 1.2 kPa
Medium VPD	28.3 ± 1.7	47.3 ± 5.4	1.9 to 2.1 kPa
High VPD	34.8 ± 2.0	31.0 ± 3.4	3.8 to 4.0 kPa

Supplementary Table S2: Table S2: Genes name, accession number, forward number, reverse number, and primer efficiency of twelve drought responsive gene transcripts measured in response to drought and evaporative demand

Gene name	Accession number	Forward primer	Reverse primer	Primer efficiency
ABA response element	GRMZM2G106622	TTCCAAGGGTTTCACAGACC	AACTATGCCAGCGATATGGG	2.10
ABA response element binding factor 1	GRMZM2G168079	TGGAGACCGTGTAAGCACAG	AGAAAAGGCTCAGGCAATCA	2.00
APETELA2(AP2/ERF) APETALA2/Ethylene Response Element Binding Factors	GRMZM2G018336	TCACAGAACAAGAACGGCAG	AAGTCAAGATCGTCGGGCTA	2.00
bHLH	GRMZM2G040349	CAGCAACAGCAACCTCAAAA	CCTTGGCTCTTGGAACTTTG	2.01
bZIP	GRMZM2G060216	ACTTCATCGGGACAGAGAGC	TTTACACCCTCCCTCACCAG	1.99
DREB	GRMZM5G889719	ACTTCATCGGGACAGAGAGC	CTGCTGCTTTGTGTTGAGGA	1.99
DREB1A	GRMZM2G124037	TCTAATTGGCGGCATAGAGG	TGGAGTTCGAGACCCTGTTC	2.00
HSF2 (Heat shock protein factor)	GRMZM2G384339	GCAAAGCTTCCACTAGCCAC	TTCGTTTCGTTTCGCTTCTT	2.01
MYB	GRMZM2G064197	CTTAGGAGGAGGCAGACACG	CTGCTGCTTTGTGTTGAGGA	1.98
NAC	GRMZM2G027309	GGCACTGGAGAGGATGAGAG	ATTCAGCAGCCATTGATTCC	2.02
WRKY	GRMZM2G063880	TCTAATTGGCGGCATAGAGG	TGGAGTTCGAGACCCTGTTC	1.89
Zinc Finger	GRMZM2G158162	AGCTGGCGATACAGTGACCT	ACAGTATTTGTGGCCTTCCG	2.02

Elongation factor 1-A (EF1-A)	NM_001112117.2	GTGAAGAATGTTGCTGTGAAGG	GCACAGCACCAGACAGATAG	1.99
Beta tubulin 7 (tub7)	NM_001112218.1	GAGGAGGAAGAAGAGGAAGAGA	CATCACCACACGAACCTCAA	2.02
